# Practices of anti-malaria pharmaceuticals inventory control system and associated challenges in public health facilities of Oromiya special zone, Amhara region, Ethiopia

**DOI:** 10.1186/s12889-021-12033-8

**Published:** 2021-11-06

**Authors:** Haile Yirga Mengesha, Getachew Moges Gebrehiwot, Birhanu Demeke Workneh, Mesfin Haile Kahissay

**Affiliations:** 1 Ethiopian Pharmaceuticals Supply Agency, Dessie hub, Dessie, Ethiopia; 2 Department of Pharmacy, College of Medicine and Health Science, Wollo University, Dessie, Ethiopia

**Keywords:** Anti-malaria pharmaceuticals, Inventory control practice, Oromia special zone, Ethiopia

## Abstract

**Background:**

Anti-malaria pharmaceuticals inventory control system helps to maintain an appropriate stock level using logistics management information system records and reports. Antimalaria pharmaceuticals are highly influenced by seasonality and demand variation. Thus, to compensate the seasonality, resupply quantities should be adjusted by multiplying the historical consumption with the Look-ahead seasonality indexes (LSI) to minimize stock-outs during the peak transmission season and overstocks (possible expiries) during off-peak seasons The purpose of this study was to assess anti-malaria pharmaceuticals inventory control practice and associated challenges in public health facilities of the Oromiya special zone, Amhara region, Ethiopia.

**Methodology:**

Facility-based cross-sectional study design employing both quantitative and qualitative methods, explanatory sequential mixed method, of data collection and analysis was used in all public health facilities in the Oromia special zone from September 1 to September 30, 2019. The study was conducted in 27 health centers and 2 hospitals, the dispensing units managing anti-malaria pharmaceuticals and data was collected using observation checklists The quantitative data were analyzed by Statistical package for social sciences using linear regression. Purposive sampling was used to select key informants and 12 in-depth interviews were conducted by the principal investigator. Thematic analysis was performed using Nvivo 11 plus and interpretation by narrative strategies.

**Results:**

The quantitative finding in this study revealed that none of the health facilities surveyed calculated months of stock and multiplied the historical consumption with look ahead seasonal indices (LSI) to forecast the upcoming year consumptions.. Average months of stock of anti-malaria pharmaceuticals were 5.32 months with the annual wastage rate of 11.32%. The point and periodic availability of anti-malaria pharmaceuticals was 72.38 and 77.03% respectively. The number of stocks out days within the previous 6 months was 41.34 days. The study also reported bin card usage (β = − 3.5, *p* = 0.04) and availability of daily dispensing register (β = − 2.7, *p* = 0.005) had statistically significant effect on anti-malaria pharmaceuticals inventory control practice. The perceived challenges attributed to the poor anti-malaria pharmaceuticals inventory control practice were lack of integrated pharmaceutical logistics system training, management support, inadequate and near expiry supply from pharmaceuticals supply agency, job dissatisfaction, and staff turnover.

**Conclusion:**

Inventory control practices for anti-malaria pharmaceuticals was poor as indicated by maximum stock level and none of the health facilities calculated months of stock and the previous consumption was not multiplied by look ahead seasonal indices to compensate the seasonal and demand variation. Efforts should be under-taken by concerned bodies to improve inventory control practice; such as training and regular follow up have to be provided to the health professionals managing anti-malaria pharmaceuticals.

**Supplementary Information:**

The online version contains supplementary material available at 10.1186/s12889-021-12033-8.

## Background

Though, reliable, equitable and efficient access to anti-malarial pharmaceuticals are needed for successful malaria diagnosis and treatment [1], an estimated 217 and 219 million cases, resulting in 451,000 and 435,000 deaths of malaria occurred globally in 2016 and 2017 respectively. Of these, 92% cases and 80% deaths occurred in 17 countries in the African region and India in 2017 [2]. Malaria still remains the leading cause of outpatient and inpatient morbidity in Ethiopia in 2017 [3].

The continuous supply of quality anti-malaria pharmaceuticals can be guaranteed through proper implementation of an appropriate logistics management information system (LMIS) and inventory control system (ICs) [4]. An ICs informs the storekeeper how to maintain an appropriate stock level of pharmaceuticals [5]. Pharmaceuticals are issued upon demand, and the stock on hand is always kept between minimum and maximum to lessen the risk of expiration and stock outs [6]. After all, an adequate quantity of health pharmaceuticals will be available at all times to meet the demand of patients and health care providers [7].

Malaria transmission has seasonal fluctuations with increased cases and demand of anti-malaria pharmaceuticals during rainy seasons. To compensate the seasonality, resupply quantities should be adjusted by multiplying the historical consumption with the Look-ahead seasonality indexes (LSI) to minimize stock-outs during the peak transmission season and overstocks (possible expiries) during off-peak seasons [8].

According to John Snow, Inc. (JSI), the identified common challenges to LMIS were poor stock record-keeping, poor consumption records and poor transaction records [9]. Medicines expiration was mainly attributed to ineffective inventory control system and stocks were ordered in excess regardless of the minimum and maximum ordering system in South Africa [10]. Availability of high-quality logistics data has been one of the greatest challenges facing the health care system in Tanzania [11]. In Uganda anti-malaria pharmaceutical security is threatened by inadequate record-keeping and information systems [12].

In Ethiopia, inventory management practice was weak by which 40.50% of the reviewed bin cards were not updated, the average accuracy rate was 28.5%, 10 (50%) of the health facilities were stocked-out of artemether/lumefantrine with a stock-out duration of 38.70 days, and high medicines wastage [13]. The problem worsens when the resupply quantity of anti-malarial pharmaceuticals is not adjusted with LSI for seasonal and demand variability which leads to frequent stock out, overstock [14], service interruption and ineffective treatment [15]. Seasonality, localized and hard to predict epidemics and poor stock visibility combine to undermine malaria commodity security in Ethiopia [16].

Limited studies has been carried out in Ethiopia and no study was conducted in this typical study area regarding anti-malaria pharmaceuticals inventory control system. Therefore, the aim of this study was to assess the practices of anti-malaria pharmaceuticals inventory control system and associated challenges in public health facilities of Oromiya special zone, Amhara region, Ethiopia. Consequently, it provides empirical implications for policy makers about current practices and track the future changes.

## Methodology

### Study area and period

The study was conducted in all public health facilities of Oromia special zone, Amhara region state, Ethiopia from September 1, to September 30, 2019. Oromia special zone is located in the eastern part of Amhara national regional state, Ethiopia. It is 331 km from Addis Ababa, the capital city of Ethiopia; and malaria endemic area. According to Oromia special zone 2019 annual report, it is administratively divided in to 7 woredas, smaller administrative units or districts, and delivering health service with 2 public hospitals, 28 health centers and 115 health posts. The total population of Oromia special zone was estimated to be 457,278 [17] .

### Study design

Facility-based cross-sectional study design employing both quantitative and qualitative methods, explanatory sequential mixed method, of data collection and analysis was conducted. The quantitative data for wastage rate, average monthly consumption and stockout days were collected retrospectively, while the data for LMIS practice, inventory accuracy rate, and stock on hand were collected at the time of visit. Phenomenological study design was used for the qualitative inquiries and the data collection was carried out by observations and interviews.

### Populations

All public health facilities, all health professionals in charge of managing anti-malaria pharmaceuticals during the data collection period and all anti-malaria pharmaceuticals managed in public health facilities of Oromiya special zone.

### Sample size determination and sampling procedures

For the quantitative study, all public health facilities in Oromia special zone were included. But one health center was excluded from the study because the facility was new and had not 6 months historical records. So, the study was conducted in 27 health centers and 2 hospitals. The dispensing units managing anti-malaria pharmaceuticals such as outpatient department (OPD) pharmacy and laboratory units were part of the study. For the qualitative part of the study, the key informants were selected by purposive sampling technique through consultation with their respective woreda logistic officers and hospital executive officers to get health professionals with comprehensive expertise in anti-malaria pharmaceuticals inventory control practices. The sample size of the study was determined by the saturation of the information provided by the key informants and 12 key informants were interviewed. The key informants were 4 store managers, 1 drug supply management officer, 1 pharmacy head and 6 dispensers.

### Data collection tools and procedures

Quantitative data was collected by 3 data collectors who were logistic officers working in the adjusting woredas outside the study area. A after half day training on the data collection instruments and processes was given prior to data collection. The principal investigator supervised the data collection process. The data was collected by reviewing all LMIS records and reports of anti-malaria pharmaceuticals using pretested facility based observation checklists prepared in English and adopted from the standard logistics indicator assessment tool (LIAT) [18] and logistics system assessment tool LSAT) [19], which was developed by USAID Deliver project. The outcome variable for this study was anti-malaria pharmaceuticals inventory control practices (see additional file 1).

The adapted assessment tools, LIAT and LSAT, include: availability of bin card, bin card usage, bin card updating, availability of RRF, RRF timelines, RRF accuracy, RRF completeness, RRF legality, RRF legibility, RRF reporting rate, resupply schedule, availability of IFRR, IFRR completeness, IFRR timeliness, IFRR legality, IFRR legibility, availability of receiving voucher, receiving voucher usage, availability of issuing voucher, issuing voucher usage, availability of IPLS SOP, technical support, availability of feedback, lead time, redistribution, staff turnover, training, staff commitment, data quality, job satisfaction, availability of pharmacy professionals, experienced staffs, management support and accountability, socio-demographic characteristics of health professionals, seasonal variation, pharmaceutical wastage, stockout and pharmaceutical availability.

The qualitative data was collected by the principal investigator using semi structured interview guide (see additional file 2) and in-depth interview and audio recorded to explore experiences of key informants until saturated. Moreover, the principal investigator took field notes of the in-depth interview. The information from the audio was transcribed verbatim. Data collection tools were first prepared in English, later translated to Amharic. The qualitative data was collected using the Amharic data collection tool and back translated to English.

### Issue of reflexivity: the principal investigator status as insider

The principal investigator status as a professional offers both strengths and limitations to the study. The principal investigator and key informants have similar profession and pharmaceutical supply chain management experience as strength to easily communicate and conduct the in-depth interview. As a limitation, He was perceived as a powerful individual due to his position as a senior pharmacy professional, It is impossible to know the extent to which his participants were truthful in the perceptions and opinions they share with him or whether they were telling him the things they think he want to hear. All of these issues concerning competing roles and perceptions related to the concept of insider bias, which has both advantages and disadvantages when conducting such a study. To mitigate the limitation, he used open-ended questions, as well as efforts made to engage key informants in informal conversations on other topics they themselves raised.

### Data processing and analysis

The quantitative data were entered and cleaned using Microsoft Excel 2010 spreadsheet and Epidata version 4.6. Thereafter, the data was exported to Statistical Package for Social Sciences (SPSS) version 20 to encode and analyze. The findings were summarized using tables and figures. The association between dependent and independent variables was tested by linear regression with 95% confidence intervals and variables with *p*-value < 0.05 were taken as statistically significant.

Qualitative data was analyzed using NVivo version 11 plus using the principles of content analysis. Early coding concurrently with data collection was conducted on audio-recorded and transcribed. Texts were read independently by the principal investigator (HYM) and another professional who speaks the local language (MHK) and codes were developed in reference to the research questions. Each of the codes were organized into higher-order conceptual themes. These individual codes and themes were discussed at group meetings until consensus was reached on basic themes and subthemes across interviews. Sections of original transcripts and key quotes considered to be illustrative of the emerging themes were translated into English to facilitate discussion with the full research team. Besides, the key informants position, profession, sex and years of experience were stated at the end of every explanation. Narrative strategies was employed for interpretation.

### Data quality assurance

For the quantitative study, the standard quantitative data collection tool was used and the facility-based observation checklists were pretested in 2 health centers outside the study area; that were not included in the study; thereafter, the sequence and lay out of the questionnaires were adjusted. Data collectors were also trained for half a day on the data collection instruments and processes of data collection and the principal investigator supervised the data collection process. Moreover, to assure the quality of the data, the questionnaire tool was properly designed and its reliability was checked by the Cronbach’s Alpha test (0.762). This was within the acceptable range for facility-based observation checklists.

For the qualitative part of the study, the semi-structured interview guide was reviewed by an expert from social and administrative pharmacy department and audio recorder were used for the interview in addition with field notes. The Amharic version was used to interview key informants. The Amharic version of the transcript was signed and returned. Conceptual framework was developed to review the theories. Mixed method, both quantitative and qualitative approaches, were employed. Moreover, multiple investigators were participated during the study. Also, the qualitative findings were shared with key informants to confirm the presentations accurately reflected their perceptions and experiences.

### Operational definitions

- **Pharmaceuticals:** drugs, chemicals, reagents, medical supplies and equipment’s used for diagnosis, prevention, and treatment of disease
- **Months of a stock**: it is defined as the usable stock on hand divided by the average monthly consumption times look ahead seasonal indices. It describes the status of stock on hand or for how long the stock on hand lasts using LMIS records and reports.
  **Months of stock** = SOH/(AMC*LSI) [20].
- **Practices of anti-malaria pharmaceuticals inventory control system:** The actual operation of maintaining the stock status between the minimum (2 months) and maximum months of stock (4 months) [20]
- **Inventory accuracy rate:** the number of items where stock record count equals physical stock count over the total number of items counted times 100 [21]
- **Point availability:** the number of usable products available in the stock at the time of review over the total number of products reviewed times 100 [21]
- **Periodic availability:** The availability of pharmaceuticals in specified period of time
- **Facility reporting rate**: the number of facilities submitting a report by a certain date over the total number of facilities required to report times 100 [21]
- **Wastage rate:** the value of the wasted pharmaceuticals over the value of total pharmaceuticals received times 100 [21]
- **RRF completeness:** All columns of the RRF are completed, reporting period and facility name is recorded.
- **RRF timeliness:** When hospitals and health centers send their RRF to the next higher level within ten (10) days following the reporting period
- **RRF accuracy:** when hospitals and health centers sent RRF without calculation error
- **High volume:** health facilities serving greater than 100 patients per day
- **Medium volume:** health facilities serving from 50 to 100 patients per day
- **Low volume:** health facilities serving less than 50 patients per day

## Results

### The socio-demographic characteristics of staffs

One hundred fifty one health professionals were managing supply chain of anti-malaria pharmaceuticals. From these, 88 (58.3%) were pharmacy professionals and 99 (65.6%) were males. With regard to their educational level, 118 (78.1%) were diploma holders. Majority of them 117 (77.5%) had work experience of less than 5 years (Table 1).

**Table 1:** Socio demographic characteristics of staffs (*n* = 151)

SN	Socio-demographic characteristics		Number	Percentage
1	Sex	Male	99	65.6
		Female	52	34.4
2	Age	20–24	76	50.3
		25–29	53	35.1
		≥ 30	22	14.6
3	Profession	Pharmacist	8	5.3
		Druggist	80	53
		Laboratory	52	34.4
		Others	11	7.3
4	Level of education	First degree	33	21.9
		Diploma	118	78.1
5	Work experience	< 5 years	117	77.5
		5–10 years	29	19.2
		> 10 years	5	3.3

A total of 12 key informants were interviewed in the qualitative study. Eight (66.7%) **of key informant** were males, the age of 6 (50%) were from 25 to 29 years. Regarding the profession, 9 (75%) were druggists. Informants included were: store managers 4(33.3%), dispensaries 6(50%), pharmacy head 1(8.3%) and supply chain officer 1(8.3%).

### Stock status of anti-malaria pharmaceuticals

A total of 11 anti-malaria pharmaceuticals were managed in the medical stores of the study health facilities. However, none of the health facilities surveyed calculated months of stock and multiplied the historical consumption with LSI, the principal investigator calculated the months of stock based on the data from health facilities. Thus, in all health facilities, all the Anti-malaria pharmaceuticals were above the maximum stock level and on average the stock on hand could serve for 5.32 months (Table 2).

**Table 2:** Stock status of anti-malaria pharmaceuticals in public health facilities of Oromia special zone, Amhara region, Ethiopia 2019 (*n* = 29)

SN	Description of items	Unit	Months of stock		
			Hospital	Health center	All health facilities
1	Artemether 20 mg-Lumfantrine120mg	Tab	4.91	5.12	5.01
2	Artesunate60mg/ml injection	Vial	3.46	5.74	4.60
3	Chloroquine 150 mg base tablet	100	0	5.32	5.32
4	Chloroquine 50 mg base/5 ml syrup	50 ml	7.58	5.35	6.46
5	Primaquine 15 mg tablet	100	5.30	5.20	5.25
6	Rapid diagnostic test (RDT)	25	NA	4.94	4.94
7	Gimsastain solution	500 ml	4.87	4.67	4.77
8	Immersion oil	100 ml	4.88	5.31	5.10
9	Methanol 95%	500 ml	0	5.55	5.55
10	Microscope slide	50	5.74	6.19	5.97
11	Blood lancet	PCS	4.35	6.74	5.54
	Average		5.14	5.47	5.32

*NA* Not applicable

**Fig. 1:**
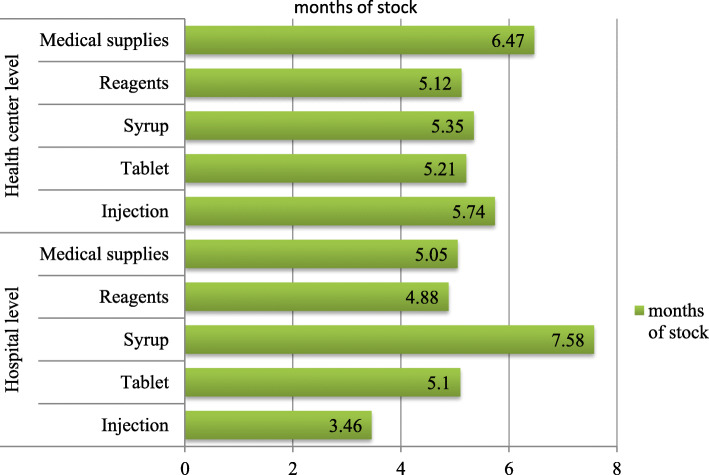
Stock status of anti-malaria pharmaceuticals by dosage forms in public health facilities of Oromia special zone, Amhara region, Ethiopia, 2019 (hospitals = 2, health centers = 27)

The average months of stock for solid, liquid dosage forms and medical supply of anti-malaria pharmaceuticals were above the maximum stock level (Fig. 1).

Ten key informants mentioned that they didn’t calculate anti-malaria pharmaceuticals months of stock and LSI hasn’t been considered.

One key informant said:

*“ … … Even though there is no similar malaria transmission throughout the year, we haven’t assessed the stock status of anti-malaria pharmaceuticals and we have never used LSI. We didn’t know for how long the stock on hand lasts. So, I don’t believe that anti-malaria pharmaceuticals have been properly requested because we have requested solely based on consumption and it might lead to stockout of artesunate injection” (Dispensary, druggist, female, 3)*

### Anti-malaria pharmaceuticals availability

The data for point availability were collected at the time of visit. The stockout days were collected in the recent last 6 months. Anti-malaria pharmaceuticals point availability in all health facilities was 72.38%. In all health facilities, the average number of stockout days, percentage of stock out days and periodic availability of anti-malaria pharmaceuticals within the previous 6 months was 41.34 days, 22.97 and 77.03% respectively (Table 3).

**Table 3:** Anti-malaria pharmaceuticals availability and stockout from January 1 to June 30, 2019, in public health facilities of Oromia special zone, Amhara region, Ethiopia (*n* = 29)

SN	Description of item	Unit	Point availability		Stock out days		Frequency of stock out	
			Hospitals	Health centers	Hospital	Health center	Hospital	Health center
1	Artemether 20 mg-Lumfantrine120mg	Tab	100.00	96.30	0.00	11.52	0.00	1.67
2	Artesunate60mg/ml injection	Vial	100.00	66.67	51.00	73.19	1.50	2.0
3	Chloroquine 150 mg base tablet	100	0.00	74.07	83.50	58.30	2.50	1.67
4	Chloroquine 50 mg base/5 ml syrup	50 ml	50.00	88.89	106.00	49.48	1.50	1.89
5	Primaquine 15 mg tablet	100	50.00	33.33	90.00	87.96	2.50	1.91
6	Rapid diagnostic test (RDT)	25	NA	96.30	NA	39.22	NA	2.27
7	Gimsastain solution	500 ml	50.00	95.65	15.00	6.74	1.00	1.25
8	Immersion oil	100 ml	100.00	86.96	7.50	34.13	1.00	1.59
9	Methanol 95%	500 ml	0.00	39.13	42.50	69.13	2.00	1.62
10	Microscope slide	50	100.00	100.00	0.00	15.22	0.00	1.77
11	Blood lancet	PCS	100.00	100.00	7.50	21.17	1.00	1.79
	Average		65.00	79.75	40.30	42.37	1.63	1.77

*NA* Not applicable

Most key informants (*n* = 11) mentioned that at least one anti-malaria pharmaceutical had been stockout last year.

One key informant said:

*“Coarteum tablet, artesunate injection and Primaquine tablet have been stockout for three, two and three months respectively, resulted in malaria epidemic and … … .. The challenges were inadequate, supply from PFSA, Dessie hub, non-consideration of LSI and the occurrence of the epidemic by itself consumed excess quantity.” (Dispensary, druggist, female, 8)*

### Anti-malaria pharmaceuticals wastage

The anti-malaria pharmaceuticals wastage rates in hospitals, health centers and in all health facilities of Oromia special zone were 11.33, 11.21 and 11.32% respectively (Table 4).

**Table 4:** Anti-malaria pharmaceuticals wastage rate in public health facilities of Oromia special zone, Amhara region, Ethiopia, from July 2018–June 2019 (*n* = 29)

Description		Beginning balance	Received	Wasted	Percentage
Health facility type	Hospital	1348.14	1192.76	284.87	11.21
	Health center	13,944.77	20,467.12	3899.22	11.33
	All health facility	15,292.91	21,659.88	4184.09	11.32
Health facility Level	High volume	4709.72	3889.85	821.49	9.55
	Medium volume	1111.48	2471.94	293.58	8.19
	Low volume	9471.71	15,298.09	3069.02	12.39

In the qualitative study, seven key informants explained that at least one anti-malaria pharmaceutical has been over stocked.

One key informant had to say:

*“Artesunate injection, coarteum tablet, and Chloroquine syrup had been overstock last year and occupied large storage space. The challenges were non-consideration of LSI during reporting and requesting anti-malaria pharmaceuticals, PFSA, Dessie hub supplied non-requested items, more than requested quantities and near expiry items.” (Dispensary, druggist, male, 6)*

### Anti-malaria pharmaceuticals inventory accuracy rate

**Fig. 2:**
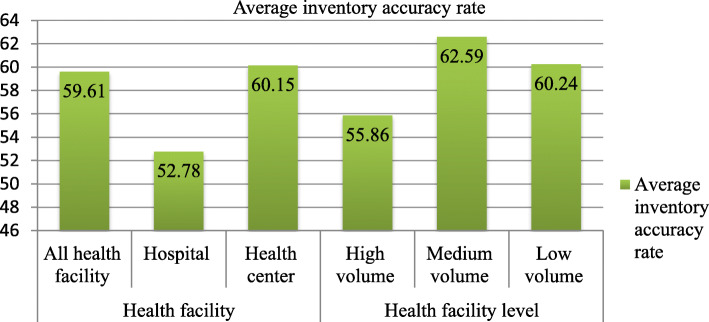
Anti-malaria pharmaceuticals inventory accuracy rate in public health facilities of Oromia special zone, Amhara region, Ethiopia, 2019 (hospitals =2, health centers = 27)

The percentages of inventory accuracy were calculated only for facilities that utilized bin cards and managed anti-malaria pharmaceuticals. It was checked by assessing the latest bin card balance with physical count on the day of the visit for the anti-malaria pharmaceuticals. The average inventory accuracy rate in all health facilities was 59.61%. The average inventory accuracy rate of anti-malaria pharmaceuticals was higher in health centers (60.15%) than hospitals (52.78%) (Fig. 2).

In the qualitative study, ten key informants explained their bin card usage.

One key informant stated:

*“ … All anti-malaria pharmaceuticals did have bin cards but not updated due to workload. As a result the physical count varies from the ending balance of the bin card.” (Dispensary, druggist, female, 3)*

### Supply chain system-related practices

The medical stores of 2 hospitals and 22 health centers were managed by pharmacy professionals. About 51.7% of store managers received supply chain training, 93.1% of the health facilities received supportive supervision within the last 6 months. Twelve (41.4%) of the store managers provided supervision to their dispensing units.

### Practices of LMIS in public health facilities

**Fig. 3:**
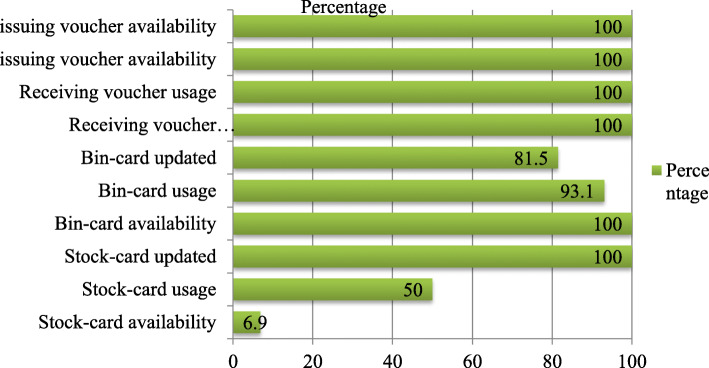
Anti-malaria pharmaceuticals stock-keeping and transaction records practice in the store of public health facilities of Oromia special zone, Amhara region, Ethiopia 2019 (*n* = 29)

All LMIS records and reports for anti-malaria pharmaceuticals were reviewed. Bin card was used and updated by 27 and 22 health facilities respectively (Fig. 3).

Most key informants (*n* = 11) mentioned that they were not using stock cards.

One key informant said:

*“Even though stock card contains a unit price and helps us to see the unit price, the stock card has not been available and used for anti-malaria pharmaceuticals yet … .moreover, since we have workload, we don’t need to bring and use stock cards, using bin card alone is enough.” (Dispensary, druggist, female, 8)*

**Fig. 4:**
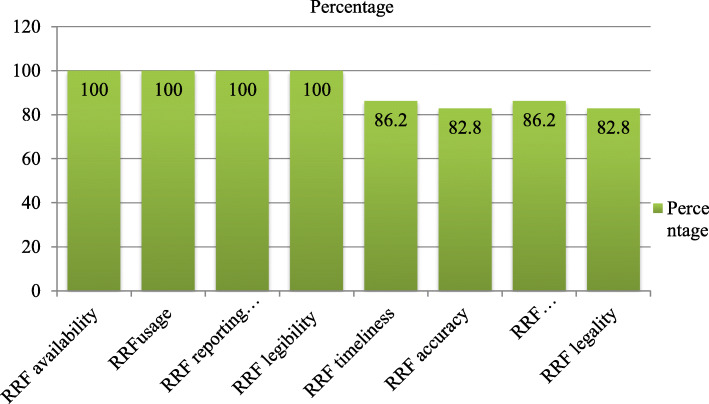
Anti-malaria pharmaceuticals transaction record practice in the stores of public health facility of Oromia special zone, Amhara region, Ethiopia, 2019 (*n* = 29)

RRF was sent every 2 months by all 29 health facilities and the reporting rate was 100%. Health facilities sent RRF timely or within 10 days after the reporting period and with data accuracy were 25 and 24 respectively. Health facilities sent RRF with complete data and legality were 25 and 24 respectively (Fig. 4).

Dispensing units resupply schedule was available in 24 health facility stores. After the mean square of OPD pharmacy and laboratory units was analyzed, the resupply schedules were followed by 22 dispensing units. None of the health facilities assessed anti-malaria pharmaceuticals stock-status and adjusted the maximum stock quantity in RFF by LSI. Only 9 health facilities redistributed overstocked anti-malaria pharmaceuticals. The lead time for anti-malaria pharmaceuticals was calculated using 3 recent RRF data. The minimum and maximum lead time was 15 and 36 days with the average lead time of 25.66 days (SD = 5.198) (Table 5).

**Table 5:** Anti-malaria pharmaceuticals stock status and system-related practices in public health facilities of Oromia special zone, Amhara region, Ethiopia, 2019 (*n* = 29)

SN	Descriptions	Number	Percentage
1	Availability of dispensing units resupply schedule	24	82.8
2	Dispensing units followed the resupply schedule	22	91.7
3	Store manager determined the resupply quantity	29	100
4	Anti-malaria pharmaceuticals stock-status assessment	0	0
5	The maximum stock quantity was adjusted by LSI in RRF	0	0
6	Redistribution of overstocked anti-malaria pharmaceuticals	9	31
7	Follow first-expire-first out	29	100
8	Damaged/expired pharmaceuticals separated from inventory	29	100
9	Emergency orders placed in the last 6 months	4	13.8
10	Number of emergency orders placed once in the last 6 months	4	100

All the key informants mentioned different types of challenges.

A key informant had to say:

*“ … . We haven’t calculated Anti-malaria pharmaceuticals months of stock and we haven’t used LSI. The lack of training or orientation was challenging’ “(Store manager, druggist, male, 2)*

Another key informant said:

*“The challenges for stockout of quinine injection, quinine tablet, artesunate injection, and Primaquine tablet was LSI non-consideration and inadequate supply from PFSA Dessie hub … … ..I didn’t have any know-how about LSI, I heard this term now for the first time from you. I didn’t take any training or orientation regarding LSI.” (Store manager, druggist, male, 2)*

**Fig. 5:**
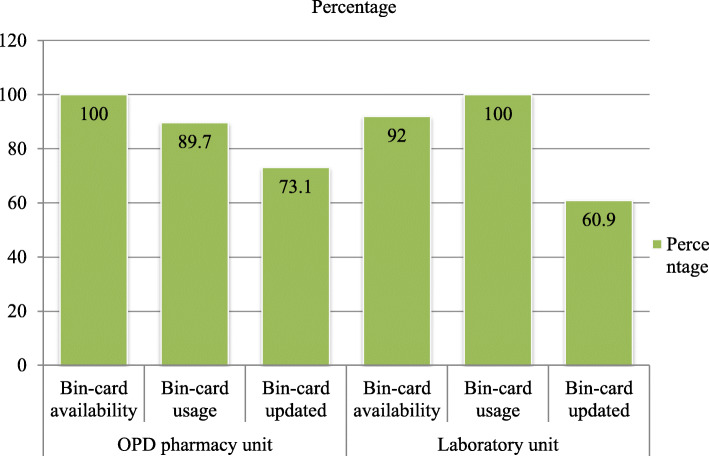
Anti-malaria pharmaceuticals stock-keeping records practice in OPD pharmacy and laboratory units in public health facilities of Oromia special zone, Amhara region, Ethiopia, 2019 (*n* = 29)

Bin card was used and regularly updated by 26 out of 29 and 19 out of 26 OPD pharmacy units of the health facilities respectively. Laboratory services were available in 25 out of 29 health facilities. Bin card was available in 23 out of 25 laboratory units and used by 23 out of 23 laboratory units. Fourteen out of 23 laboratory units updated their bin cards regularly (Fig. 5).

**Fig. 6:**
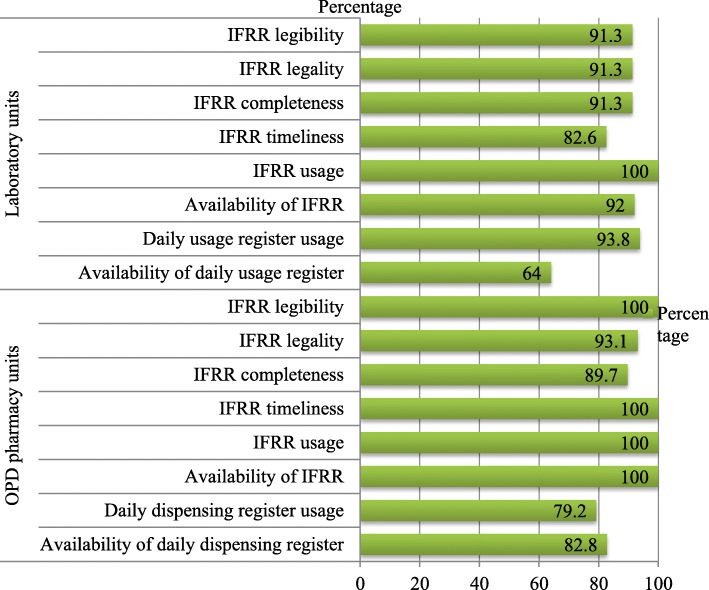
Anti-malaria pharmaceuticals transaction and consumption records practice in OPD pharmacy and laboratory units in public health facilities of Oromia special zone, Amhara region, Ethiopia, 2019 (*n* = 29)

The study investigated that IFRR was available, used, sent timely and legible in all OPD pharmacy units. Whereas the IFRR was legal and complete in 27 and 26 out of 29 OPD pharmacy dispensing units of the health facilities respectively. IFRR was available in 23 out of 25 laboratory units and used by 23 out of 23 laboratory units. Laboratory units that sent IFRR timely were 19 out of 23 laboratory units. In addition, the IFRR was complete, legible and legal in 21 out of 23 laboratory units (Fig. 6).

Bin card usage (β = − 3.5, *p* = 0.04) and availability of daily dispensing register (β = − 2.7, *p* = 0.005) have statistically significant effect on anti-malaria pharmaceuticals inventory control practice (Table 6).

**Table 6:** Multiple linear regression output of anti-malaria pharmaceuticals inventory control practice in public health facilities of Oromia special zone, Amhara region, Ethiopia, 2019, (*n* = 29)

SN	Descriptions	*P* value	β (95% CI)
1	Bin card usage	0.04	−3.5(−6.9- -0.2)
2	Availability of daily dispensing register	0.005	−2.7(−4.4- -1)
3	Availability of IPLS SOP	0.717	0.3(−1.3–1.9)
4	Availability of stock card	0.320	−1.5(−4.9–1.8)
5	RRF accuracy	0.672	0.35(−1.5–2.2)

When health facilities that didn’t use bin card for anti-malaria pharmaceuticals, started using bin card the months of stock will be changed by − 3.5. When the health facilities that didn’t have daily dispensing register, try to avail daily dispensing register the months of stock will be changed by − 2.7.

## Discussion

The ultimate goal of anti-malaria pharmaceuticals inventory control system is to keep the stock on hand between minimum and maximum. This can be achieved by assessing stock status and using look ahead seasonal indices. So, the present study focused on the practice of anti-malaria pharmaceuticals inventory control system and associated challenges in public health facilities of Oromia special zone, Amhara region, Ethiopia. However, none of the health facilities surveyed calculated months of stock and multiplied the historical consumption with LSI, the principal investigator calculated the months of stock based on the data from health facilities.

In the present study, the anti-malaria pharmaceutical’s average months of stock were 5.32 months. This was above the maximum stock level or overstock where the minimum and the maximum stock levels are below 2 and above 4 months respectively [20]. Similar study conducted in North Wollo and Waghemira zones, Ethiopia, reported poor implementation of the inventory control system [22].

The study also revealed that, artemether 20 mg-Lumfantrine120mg and Artesunate60mg/ml injection were above maximum stock levels with average months of stock 5.01 and 4.6 months respectively, which are similar to the study conducted in Uganda where stock levels of artesunate injectable and the four artemether/lumefantrine pack sizes were above the maximum months of stock level [23]. A similar finding also reported in Mali where all health facilities had above the maximum level of artesunate60mg/ml injection and higher than RDT where 37.5% of the health facilities had maximum stock level [24]. The differences might be due to the difference in the geographic location of the area and study period.

In this study, the average months of stock for anti-malaria medical supply at the health facility level was 5.76 months which was overstock and the study conducted in Saint-Paul hospital revealed that the share of medical supplies expenditure was 45% which is much higher than the shares of medicines and laboratory reagents [25]. However, the quantitative finding in this study revealed that none of the health facilities surveyed calculated months of stock and multiplied the historical consumption with LSI, the qualitative finding in this study also revealed that none of the key informants assessed anti-malaria pharmaceuticals stock status and never used LSI. Informants requested based on consumption alone. The reasons could be due to a lack of training and inadequate supportive supervision provided by higher levels.

The anti-malaria pharmaceuticals average point availability was 65% and 79.75 in hospitals and health centers respectively. This finding was lower and higher than the national survey conducted in Ethiopia where the average point availability in hospitals and health centers was 92.5 and 76.8% respectively [26]. The anti-malaria pharmaceuticals average point availability in this study was 72.38% in all health facilities, which was lower than the study conducted by Daniel where the pharmaceuticals point availability was 97.5% [27]. Moreover in this study, the point availability of artesunate injection and RDT were 83.34 and 96.30% respectively which were higher than the study conducted in Mali where the point availability of artesunate injection and RDT were 68.35 and 94.94% respectively [24]. The difference could be because of the type and quantity of medicines selected for study as well as the type of facility involved in the study. The point availability of this finding is lower than the national target found in the health sector transformation plan of Ethiopia which was intended to increase the availability of essential drugs to 100% [28].

In the present study, the average number of stockout days of anti-malaria pharmaceuticals at health centers in the previous 6 months was 42.37 days (23.54%) which is slightly lower than the study conducted in Guji zone where the average stockout days in the previous 6 months in health centers were 42.78 days (23.77%) and the periodic availability within the previous 6 months in this study was 76.46%, which is higher than the study conducted in the Guji zone, Oromia regional state where the periodic availability was 76.23% in health centers [29]. The difference might be due to the types of pharmaceuticals included in the study.

The average number of stockout days for Artemether 20 mg-Lumfantrine120mg tablet in the present study within the last 6 months was 5.76 days which is lower than the study conducted in East Shewa Zone where the number of the stockout days for Artemether20mg-Lumfantrine120mg tablet in the last 6 months was 38.70 days [13]. The key informants in the present study revealed that Coarteum tablet, Artesunate injection and Primaquine tablet have been stockout.

In the current study, the annual anti-malaria pharmaceuticals average wastage rate was 11.21, 11.33 and 11.32% at hospitals, health centers and in all health facilities respectively. These findings were higher than the study conducted in the south west Shoa zone where the wastage rate was 6, 8.5 and 7.5% in hospitals, health centers and in all health facilities respectively [30]. These findings were also higher than the national target in Ethiopia that was intended to reduce the wastage rate to less than 2% by 2015/16–2019/20 [28]. The qualitative finding in this study revealed that Artesunate injection, coarteum tablet, and Chloroquine syrup has been overstock last year and occupied large storage space. The reason for high wastage rate might be non-considering LSI during reporting and ordering, poor management support, poor quantification and lack of accountability, supply of non-requested, over requested and near expiry items from PFSA, Dessie hub.

The present study revealed that the average inventory accuracy rate of anti-malaria pharmaceuticals in hospitals and health centers were 52.78 and 60.15% respectively, which is higher than the national IPLS survey with average inventory accuracy rate at hospitals (49%) and health centers (59.4%) [26], but lower than the national target of achieving 95% [31]. The qualitative finding revealed that the physical counts varied from the ending balance found in the bin cards. The difference might be because of the difference in the sample size of the study and pharmaceuticals included in the study.

In this study, 82.8% of store managers were pharmacy professionals which was higher than the study conducted in North *Wollo* and *Waghimera* zone, where 64.6% of the store managers were appropriate professionals [22] and lower than the national IPLS survey, where 85% of the health facilities had pharmacy professionals in their staff [26]. The difference could be due to the difference in the study period and area.

The store managers who received supply chain training in the present study were 51.7% which is lower than the study conducted Workneh, where 90.2% of store managers received training consisting inventory control system [22] and the national IPLS survey in which 76% of the health facilities received IPLS training [26]. The differences found might be due to trained staff attrition.

In this study, 93.1% of the health facilities received supportive supervision within the last 6 months, which was higher than the national IPLS survey, where 88% of the health facilities received supportive supervision within the last 6 months [32] and lower than the study conducted in public health facilities of East Wollega Zone, where 95.56% of the study facilities had received supportive supervision [33]. The differences found might be due to the difference in the sample size of study. This study revealed that IPLS SOP is available in 68.97% of the health facilities which is higher than the national IPLS survey where the IPLS SOP availability was 49.2% [26] and lower than the study conducted in East Shewa Zone where the IPLS SOP availability was 100% [13].

In the present study 50% of the health facilities used stock card, which is lower than the study conducted by Mudzteba, where 87.5% of the health facilities used stock card [34]. The key informants of this study also revealed that the stock card has not been available and used for anti-malaria pharmaceuticals yet. The reasons could be a poor attitude among professionals regarding its usage. Bin card usage in the present study was 93.1%, which is higher than the national IPLS survey where the bin card usage was 83% [26]. The findings of this study indicated 81.5% of health facilities updated bin cards which is higher than the study conducted in East Shewa Zone where 59.5% of the bin cards was updated [13] and in health centers of Addis Ababa where 69.5% of the bin cards were updated [34]. The difference could be because of the type of medicines selected for the study.

The RRF reporting rate in the current study was 100%, which is higher than the study conducted in East Wollega Zone where the RRF reporting rate was 97% [33]. This study revealed that 86.2% of the health facilities surveyed sent their RRF timely, which is higher than the study conducted in East Wollega Zone where 69.4% of the health facilities sent their RRF timely [33] and lower than Mali where 91.14% of the facilities submit reports on time [24]. In this study 86.2% of the RRF sent was complete, which is lower than the study conducted in East Wollega Zone where 97.8% of the RRF sent was complete [33]. In the current study, 82.8% of the health facilities sent accurate RRF data, which is higher than the study conducted in East Wollega Zone, where 64.6% of the health facilities sent an accurate report [33]. The variation could be due to differences in the competency of professionals, management support and follow up.

The current study revealed that the lead time for anti-malaria pharmaceuticals ranges from 15 to 36 days, which is higher than the study conducted by Mudzteba where the majority of the health centers took 1 to 2 weeks to receive pharmaceuticals from PFSA after an order had been, initiated [34]. The difference might be due to the distance between the supplier and the health facilities.

In the current study, 89.7% of the OPD pharmacy units used bin cards, which is lower than the study conducted in East Shewa Zone where OPD pharmacy of all health facilities used bin cards [13]. In this study, IFRR availability and usage for anti-malaria pharmaceuticals were 100% in OPD pharmacy units of the health facilities studied, similar to the study conducted in health facilities of East Shewa Zone, where IFRR was available and used by all the OPD pharmacy units of studied health facilities [13]. The reasons for the differences might be due to the difference in study time and the health professionals’ knowledge and commitment.

Bin card usage (β = − 3.5, *p* = 0.04) and availability of daily dispensing register (β = − 2.7, *p* = 0.005) have statistically significant effect on inventory control practice of anti-malaria pharmaceuticals. When health facilities that didn’t use bin card for anti-malaria pharmaceuticals, started using bin card the months of stock will be changed by − 3.5. When the health facilities that didn’t have daily dispensing register, try to avail daily dispensing register the months of stock will be changed by − 2.7.

### Practical implications

Bin card usage is critical because the essential data items required for calculating months of stock such as average monthly consumption and stock on hand can be extracted from bin card. Thus, the bin card is a source of data for assessing anti-malaria pharmaceuticals inventory control practice. In addition, reports are generated using the data available in the bin cards. The availability of daily dispensing register helps the dispensing units to register daily dispensed pharmaceuticals and it will be the most important source of actual consumption data required for assessing the months of stock. Therefore, the daily dispensing register is an actual source of data for assessing anti-malaria pharmaceuticals inventory control practice.

In the current study, the anti-malaria pharmaceuticals surveyed were above the maximum stock level and the annual anti-malaria pharmaceuticals wastage rate was above the national standard value. These findings imply that there are misuses of these pharmaceuticals, which leads to inappropriate uses of the facilities limited resources and affect health care delivery system at large. Moreover, anti- malaria pharmaceuticals inventory control practices would have public health, economic and clinical impact if there is shortage or overage of pharmaceuticals due to inappropriate practices.

### Strengths and limitations of the study

The strengths of this study include that this study employed both quantitative and qualitative methods to supplement each other. The limitation of this study was that anti-malaria pharmaceuticals average monthly consumption was calculated using proxy (issue) data from the medical store to the dispensing units by observing the bin card or model 22 rather than the actual consumption dispensed from the dispensing units to clients.

## Conclusion

Anti-malaria pharmaceuticals inventory control practice in the surveyed health facilities was poor where none of the surveyed health facilities calculated months of stock and not considered multiplication of the previous consumption by LSI to compensate for the seasonality and demand variation. At the time of visit, all the anti-malaria pharmaceuticals surveyed were above the maximum stock level and Chloroquine 50 mg base/5 ml syrup was above maximum stock level with the highest months of stock. The annual anti-malaria pharmaceuticals wastage rate was above the national standard value. Anti-malaria pharmaceuticals point availability and periodic availability was below the national target. The challenges attributed to the poor anti-malaria pharmaceuticals inventory control practice were lack of IPLS training, inadequate, near expiry supply from PFSA and not consideration of LSI. Efforts should be under-taken by Ethiopian Pharmaceuticals Supply Agency (EPSA), federal ministry of health/Amhara regional health bureau/Oromia Special Zone, Woreda health office and health facilities to improve inventory control practice; such as IPLS training, LSI orientation and regular follow up have to be provided to the health professionals managing anti-malaria pharmaceuticals.

## Supplementary Information

**Additional file 1.**

**Additional file 2.**

**Additional file 3.**

## Data Availability

The datasets used and/or analysed during the current study are available from the corresponding author on reasonable request.

## Abbreviations

SOH: Stock on hand
AMC: Average monthly consumption
LSI: Look ahead seasonal indices
PFSA: Pharmaceuticals fund and supply agency
EPSA: Ethiopian pharmaceuticals supply agency
HCMIS: Health commodity management information system
LMIS: Logistics management information system
ICs: Inventory control system
RRF: Report and requisition form
IFRR: Internal facility report and resupply form
IPLS: Integrated Pharmaceuticals logistic system
SOP: Standard operating procedure
LIAT: Logistics indicator assessment tool
LSAT: Logistics system assessment tool
OPD: Outpatient department
RDT: Rapid diagnostic test
SIAPS: Systems for improved access to pharmaceuticals and services
SD: Standard deviation
SPSS: Statistical package for social sciences
WHO: World health organization
USAID: United states agency for international development
AIDS: Acquired immune deficiency syndrome
FMOH: Federal ministry of health
MMV: Medicines for malaria venture
JSI: John Snow, Inc.
