# Supplementary material for: Practices of anti-malaria pharmaceuticals inventory control system and associated challenges in public health facilities of Oromiya special zone, Amhara region, Ethiopia
Source: BMC Public Health. 2021 Nov 6;21:2026. doi: 10.1186/s12889-021-12033-8 (PMC8572494; doi:10.1186/s12889-021-12033-8)
Supplement: Supplementary file 1 — Additional file 1. [file 12889_2021_12033_MOESM1_ESM.docx]

**Data collection tools**

Section 1: Assessment of anti-malaria pharmaceuticals inventory control practice in public health facilities

1. Background information about health facility

| SN | Description | Facility identification |
| --- | --- | --- |
| 1 | Name of the health facility |  |
| 2 | Code of the health facility |  |
| 3 | Type of health facility | Hospital ………….....……..  1 |
|  |  | Health center ………..….…  2 |
| 4 | Level of health facility based on patient load | Low Volume...………...…..  1 |
|  |  | Medium Volume …….........  2 |
|  |  | High Volume …...……........  3 |
| 5 | Type of health facility based on automation | HCMIS site ………….….....  1 |
|  |  | Non HCMIS site………......  2 |

1. Data collection sheet for anti-malaria pharmaceuticals wastage for 2011 EFY

| SN | Description of item | Beginning balance  (ETB) | Received (ETB) | Wasted (ETB) | Remark |
| --- | --- | --- | --- | --- | --- |
| 1 | Artemether 20mg-Lumfantrine120mg 6x1 |  |  |  |  |
| 2 | Artemether 20mg-Lumfantrine120mg 6x2 |  |  |  |  |
| 3 | Artemether 20mg-Lumfantrine120mg 6x3 |  |  |  |  |
| 4 | Artemether 20mg-Lumfantrine120mg 6x4 |  |  |  |  |
| 5 | Artesunate 60mg/ml injection |  |  |  |  |
| 6 | Quinine300mg/1ml injection |  |  |  |  |
| 7 | Quinine 300mg tablet |  |  |  |  |
| 8 | Chloroquine 150mg base tablet |  |  |  |  |

| 9 | Chloroquine 50mg base/5ml syrup |  |  |  |  |
| --- | --- | --- | --- | --- | --- |
| 10 | Primaquine 15mg tablet |  |  |  |  |
| 11 | Rapid diagnostic test kits (RDT) |  |  |  |  |
| 12 | Gimsastain solution 500ml |  |  |  |  |
| 13 | Immersion oil 100ml |  |  |  |  |
| 14 | Methanol 95% 500ml |  |  |  |  |
| 15 | Microscope slide |  |  |  |  |
| 16 | Blood lancet |  |  |  |  |
| 17 | Microscope |  |  |  |  |

1. Data collection sheet for anti-malaria pharmaceuticals availability in public health facilities

| SN | Description of item | Unit | BC available (Y/N) | BC updated (Y/N) | Number of months of data available | Balance on BC | Stock out most recent 6 months (Y/N) | Number of stock outs | Total number stock out days | Issued in recent 3 months | Usable stock today |
| --- | --- | --- | --- | --- | --- | --- | --- | --- | --- | --- | --- |
| 1 | Artemether 20mg-Lumfantrine120mg | 1X6 |  |  |  |  |  |  |  |  |  |
| 2 | Artemether 20mg-Lumfantrine120mg | 2X6 |  |  |  |  |  |  |  |  |  |
| 3 | Artemether 20mg-Lumfantrine120mg | 3X6 |  |  |  |  |  |  |  |  |  |
| 4 | Artemether 20mg-Lumfantrine120mg | 4X6 |  |  |  |  |  |  |  |  |  |
| 5 | Artesunate 60mg/ml injection | Vial |  |  |  |  |  |  |  |  |  |
| 6 | Quinine300mg/1ml injection | Vial |  |  |  |  |  |  |  |  |  |
| 7 | Quinine 300mg tablet | 100 |  |  |  |  |  |  |  |  |  |
| 8 | Chloroquine 150mg base tablet | 100 |  |  |  |  |  |  |  |  |  |
| 9 | Chloroquine 50mg base/5ml syrup | 60ml |  |  |  |  |  |  |  |  |  |
| 10 | Primaquine 15mg tablet | 100 |  |  |  |  |  |  |  |  |  |
| 11 | Rapid diagnostic test kits (RDT) | 25 |  |  |  |  |  |  |  |  |  |
| 12 | Gimsastain solution | 500ml |  |  |  |  |  |  |  |  |  |
| 13 | Immersion oil | 100ml |  |  |  |  |  |  |  |  |  |

| 14 | Methanol 95% | 500ml |  |  |  |  |  |  |  |  |  |
| --- | --- | --- | --- | --- | --- | --- | --- | --- | --- | --- | --- |
| 15 | Blood lancet | 50 |  |  |  |  |  |  |  |  |  |
| 16 | Microscope slide | 50 |  |  |  |  |  |  |  |  |  |
| 17 | Microscope | each |  |  |  |  |  |  |  |  |  |

1. Checklist for assessment of pharmaceutical supply chain systems in public health facilities. The following statements are intended to assess the supply chain systems in public health facilities

| SN | Description | | Remark |
| --- | --- | --- | --- |
| 1 | The medical store is managed by | Pharmacy 1  Nurse 2  Other (specify) |  |
| 2 | Number of store manager | One 1  Two 2 |  |
| 3 | Store manager received supply chain and/or  other related training in the last 6 months | Yes 1  No 2 | If no skip to 5 |
| 4 | Types of training the store manager received |  |  |
| 5 | The health facility receive supportive  supervision | Yes 1  No 2 | If no skip to  next part |
| 6 | Frequency of supportive supervision | Every month 1  Every quarter 2  Every six month 3  Other(Specify) |  |
| 7 | The supportive supervision was provided by | RHB/ZHD/WoHO... 1  PFSA 2  Partners 3 |  |
| 8 | The health facility receive feedback from  higher levels | Yes 1  No 2 |  |
| 9 | Feedback mechanisms | Written 1  Verbal feedback 2 |  |

|  |  | Both feedbacks. 3 |  |
| --- | --- | --- | --- |

1. Checklist for anti-malaria pharmaceuticals inventory control practice in public health facilities

These statements require observation and document review to evaluate the practice of antimalaria pharmaceuticals inventory control. First, communicate the in-charge person and then confirm by observation and document review.

- 1. Health facility store

| SN | Inventory control system practice | | Remark |
| --- | --- | --- | --- |
| 1 | Availability of IPLS standard operating  procedure in the facility | Yes 1  No 2 |  |
| 2 | Availability of stock card | Yes 1  No 2 | If no skip to 5 |
| 3 | Stock card usage | Yes 1  No 2 | If no skip to 5 |
| 4 | Stock card regularly updated | Yes 1  No 2 |  |
| 5 | Availability of bin card | Yes 1  No 2 | If no skip to 9 |
| 6 | Bin card usage | Yes 1  No 2 | If no skip to 9 |
| 7 | Bin card regularly updated | Yes 1  No 2 |  |
| 8 | The bin card included the 3 essential data items | Yes 1  No 2 |  |
| 9 | Availability of receiving voucher ( model-19) | Yes 1  No 2 | If no skip to 11 |
| 10 | Receiving voucher ( model-19) usage | Yes 1  No 2 |  |
| 11 | Availability of issuing voucher ( model-22) | Yes 1  No 2 | If no skip to 13 |

| 12 | Issuing voucher ( model-22) usage | Yes 1  No 2 |  |
| --- | --- | --- | --- |
| 13 | Availability of RRF | Yes 1  No 2 | If no skip to 24 |
| 14 | RRF usage | Yes 1  No 2 | If no skip to 24 |
| 15 | The RRF included the three essential data items | Yes 1  No 2 |  |
| 16 | The facility sent RRF every two months | Yes 1  No 2 |  |
| 17 | Timeliness of RRF | Yes 1  No 2 | If yes skip to 19 |
| 18 | Number of days after reporting period |  |  |
| 19 | The accuracy of the RRF (Calculation) | Yes 1  No 2 |  |
| 20 | Completeness of RRF | |  |
|  | Completeness of reporting period | Yes 1  No 2 |  |
|  | Facility name recorded | Yes 1  No 2 |  |
|  | All columns completed | Yes 1  No 2 |  |
| 21 | The legality of the RRF | |  |
|  | Completed by signature | Yes 1  No 2 |  |
|  | Verified by signature | Yes 1  No 2 |  |
|  | Approved by signature | Yes 1  No 2 |  |
|  | Availability of facility stamp | Yes 1 |  |

|  |  | No 2 |  |
| --- | --- | --- | --- |
| 22 | Legibility of the RRF | Yes 1  No 2 |  |
| 23 | Number of days the recent RRF was sent  following the reporting period |  |  |
| 24 | Availability of dispensing units resupply  schedule | Yes 1  No 2 | If no skip to 26 |
| 25 | Dispensing units follow resupply schedule |  |  |
|  | OPD pharmacy unit | Yes 1  No 2 |  |
|  | Laboratory unit | Yes 1  No 2 |  |
| 26 | The store manager determine resupply quantity  of dispensing units | Yes 1  No 2 |  |
| 27 | Anti-malaria pharmaceuticals stock status  assessment | Yes 1  No 2 | If no skip to 29 |
| 28 | Look ahead seasonal indices (LSI) used during  stock status assessment | Yes 1  No 2 |  |
| 29 | Adjust the maximum stock quantity by LSI in  preparing RRF | Yes 1  No 2 |  |
| 30 | Source of antimalaria pharmaceuticals | PFSA 1  RHB/ZHD/Woreda 2  Partners 3 |  |
| 31 | Average length of time between ordering and  receiving of anti-malaria pharmaceuticals (3 consecutive RRF) |  |  |
| 32 | written provisions for the redistribution of over-  stocked anti-malaria pharmaceuticals | Yes 1  No 2 |  |
| 33 | Follow first-to-expire, first-out (FEFO)  inventory control procedures | Yes 1  No 2 |  |

| 34 | Damaged/expired pharmaceuticals are  physically separated from inventory | Yes 1  No 2 |  |
| --- | --- | --- | --- |
| 35 | Emergency orders placed in the last 6 months | Yes 1  No 2 | If no skip to 37 |
| 36 | Number of emergency orders placed in the last  six months |  |  |
| 37 | Provide supportive supervision to dispensing  units | Yes 1  No 2 | If no skip to next  part |
| 38 | The frequency of supportive supervision  provided by the store manager |  |  |
| 39 | The store manager provides feedback | Yes 1  No 2 | If no skip to next  part |
| 40 | Feedback mechanisms | Verbal… 1  Written… 2  Both… 3 |  |

- 1. OPD pharmacy unit

| SN | Inventory control system practice | | Remark |
| --- | --- | --- | --- |
| 1 | Availability of bin card | Yes 1  No 2 | If no skip to 4 |
| 2 | Bin card usage | Yes 1  No 2 | If no skip to 4 |
| 3 | Bin card regularly updated | Yes 1  No 2 |  |
| 4 | Availability of daily dispensing register | Yes 1  No 2 | If no skip to 6 |
| 5 | Daily dispensing register usage | Yes 1  No 2 |  |
| 6 | Availability of IFRR | Yes 1  No 2 | If no skip to next  part |
| 7 | IFRR usage | Yes 1  No 2 | If no skip to next  part |
| 8 | Timeliness of IFRR | Yes 1  No 2 |  |
| 9 | Completeness of IFRR | |  |
|  | Completeness of reporting period | Yes 1  No 2 |  |
|  | Dispensing unit name recorded | Yes 1  No 2 |  |
|  | All columns of dispensing unit completed | Yes 1  No 2 |  |
| 10 | Legality of IFRR | |  |
|  | Completed by dispensing unit has signature | Yes 1  No 2 |  |
|  | Verified by signature | Yes 1  No 2 |  |
|  | Approved by signature | Yes 1 |  |

|  |  | No 2 |  |
| --- | --- | --- | --- |
| 11 | Legibility of IFRR | Yes 1  No 2 |  |

- 1. Laboratory unit

| SN | Inventory control system practice | | Remark |
| --- | --- | --- | --- |
| 1 | Availability of laboratory service | Yes 1  No 2 | If no skip to the  next part |
| 2 | Availability of bin card | Yes 1  No 2 | If no skip to 4 |
| 3 | Bin card usage | Yes 1  No 2 | If no skip to 4 |
| 4 | Bin card regularly updated | Yes 1  No 2 |  |
| 5 | Availability of daily usage register | Yes 1  No 2 | If no skip to 6 |
| 6 | Daily usage register usage | Yes 1  No 2 |  |
| 7 | Availability of IFRR | Yes 1  No 2 | If no skip to next  part |
| 8 | IFRR usage | Yes 1  No 2 | If no skip to next  part |
| 9 | Timeliness of IFRR | Yes 1  No 2 |  |
| 10 | Completeness of IFRR | |  |
|  | Completeness of reporting period | Yes 1  No 2 |  |
|  | Dispensing unit name recorded | Yes 1  No 2 |  |
|  | All columns of dispensing unit completed | Yes 1  No 2 |  |
| 11 | Legality of IFRR | |  |

|  | Completed by dispensing unit has signature | Yes 1  No 2 |  |
| --- | --- | --- | --- |
|  | Verified by signature | Yes 1  No 2 |  |
|  | Approved by signature | Yes 1  No 2 |  |
| 12 | Legibility of IFRR | Yes 1  No 2 |  |

**Socio-demographic Characteristics of Respondents**

For each statement, please encircle one number which best describes you

| SN | Description | | | |
| --- | --- | --- | --- | --- |
| 1 | Sex | Male 1  …….………………..… 2 | Female | |
| 2 | Age (Year) |  | | |
| 3 | Work experience (Years) |  | | |
| 4 | Salary |  | | |
| 5 | Profession | Pharmacist 1 | Nurse diploma 6 | |
|  |  | Druggist 2  7 | Health officer ……….…..… | |
|  |  | Laboratory degree 3  8 | Midwifery degree ……. …... | |
|  |  | Laboratory diploma 4  …….…..9 | Midwifery diploma | |
|  |  | Nurse degree 5 | Other (Please specify) | |
| 6 | Have you ever taken supply  chain and other related training | Yes 1  No 2 | | If no skip to  next part |
| 7 | Types of training received |  | | |

1. Perceived challenges related to anti-malaria pharmaceuticals inventory control system practices

Please give your answer by identifying challenges related to anti-malaria pharmaceuticals inventory control practices by encircling the numbers provided after each question

| SN | Questions | Level of responses | | | | |
| --- | --- | --- | --- | --- | --- | --- |
|  |  | **Strongly disagree** | **Disagree** | **Neutral** | **Agree** | **Strongly agree** |
| 1 | Non-availability of IPLS standard operating procedure | 1 | 2 | 3 | 4 | 5 |
| 2 | Non-availability of recording formats | 1 | 2 | 3 | 4 | 5 |

| 3 | Non-updating of recording formats | 1 | 2 | 3 | 4 | 5 |
| --- | --- | --- | --- | --- | --- | --- |
| 4 | Non-availability of reporting formats | 1 | 2 | 3 | 4 | 5 |
| 5 | Inaccurate, incomplete and late RRF | 1 | 2 | 3 | 4 | 5 |
| 6 | Non-consideration of LSI during reporting and ordering | 1 | 2 | 3 | 4 | 5 |
| 7 | Inadequate supply from PFSA | 1 | 2 | 3 | 4 | 5 |
| 8 | Near expiry supply from PFSA | 1 | 2 | 3 | 4 | 5 |
| 9 | DUs not following resupply schedule | 1 | 2 | 3 | 4 | 5 |
| 10 | Frequent emergency orders | 1 | 2 | 3 | 4 | 5 |
| 11 | Overstock of anti-malaria pharmaceuticals | 1 | 2 | 3 | 4 | 5 |
| 12 | Stock out of anti-malaria pharmaceuticals | 1 | 2 | 3 | 4 | 5 |
| 13 | No redistribution of overstocked anti-malaria pharmaceuticals | 1 | 2 | 3 | 4 | 5 |
| 14 | Expired, damaged and lost anti- malaria pharmaceuticals | 1 | 2 | 3 | 4 | 5 |
| 15 | Damaged and expired anti-malaria  pharmaceuticals not stored separately | 1 | 2 | 3 | 4 | 5 |
| 16 | Non-timely disposal of anti-malaria pharmaceutical wastes | 1 | 2 | 3 | 4 | 5 |
| 17 | Non-adherence to standard treatment guideline | 1 | 2 | 3 | 4 | 5 |
| 18 | Lack of regular supportive supervision | 1 | 2 | 3 | 4 | 5 |
| 19 | Lack of Phar,cal supply chain performance review meeting | 1 | 2 | 3 | 4 | 5 |
| 20 | Lack of feedback provision | 1 | 2 | 3 | 4 | 5 |
| 21 | Lack of management support | 1 | 2 | 3 | 4 | 5 |

| 22 | Shortage of pharmacy professionals | 1 | 2 | 3 | 4 | 5 |
| --- | --- | --- | --- | --- | --- | --- |
| 23 | Non- trained staff | 1 | 2 | 3 | 4 | 5 |
| 24 | Non- experienced staff | 1 | 2 | 3 | 4 | 5 |
| 25 | Lack of skill transfer | 1 | 2 | 3 | 4 | 5 |
| 26 | Staff turnover | 1 | 2 | 3 | 4 | 5 |
| 27 | Lack of staff commitment | 1 | 2 | 3 | 4 | 5 |
| 28 | Job dissatisfaction | 1 | 2 | 3 | 4 | 5 |
| 29 | Lack of accountability | 1 | 2 | 3 | 4 | 5 |
