# Supplementary material for: Practices of anti-malaria pharmaceuticals inventory control system and associated challenges in public health facilities of Oromiya special zone, Amhara region, Ethiopia
Source: BMC Public Health. 2021 Nov 6;21:2026. doi: 10.1186/s12889-021-12033-8 (PMC8572494; doi:10.1186/s12889-021-12033-8)
Supplement: Supplementary file 2 — Additional file 2. [file 12889_2021_12033_MOESM2_ESM.docx]

**Interview guiding for key informant interview (English version)**

1. Background information of the key informant

1. Age ……………………………………………

2. Sex ……………………………………………

1. Level of education ……………………………

4. Profession ……………………………………

1. Work experience ……………………………..
2. The current position in the health facility…………..
3. **Guiding questions for an in-depth interview with health professionals managing anti-malaria pharmaceuticals**
4. How do you evaluate the anti-malaria pharmaceuticals inventory control system practice? Explain? concerning
   1. Availability of LMIS formats
   2. Using LMIS formats (recording and reporting)
   3. Stock status assessment
5. What are the challenges related to anti-malaria pharmaceuticals inventory control practice? Explain? concerning
   1. Availability of LMIS formats
   2. Using LMIS formats (recording and reporting)
   3. Stock status assessment
6. Did you encounter with stock-out of anti-malaria pharmaceuticals? If yes, which types of pharmaceuticals were stocked out? What were the challenges for stock out?
7. Did you encounter with an overstock of anti-malaria pharmaceuticals? If yes, which types of pharmaceuticals were overstocked? What were the challenges for overstock?
8. What is the importance of the inventory control system and what is new in the case of anti- malaria pharmaceuticals? (Related to months of stock and LSI).

**Thank you for your time and cooperation**
