# Supplementary material for: Practices of anti-malaria pharmaceuticals inventory control system and associated challenges in public health facilities of Oromiya special zone, Amhara region, Ethiopia
Source: BMC Public Health. 2021 Nov 6;21:2026. doi: 10.1186/s12889-021-12033-8 (PMC8572494; doi:10.1186/s12889-021-12033-8)

**written consent form for self-administered questionnaire on the perceived challenges on anti-malaria pharmaceuticals inventory control system**

**Good day,** My name is Haile Yirga, from Wollo University, pharmacy department. I am doing my thesis for the partial fulfillment of a master’s degree in social and administrative pharmacy. My study is designed to **assess** the **practices of antimalaria pharmaceuticals inventory control system and associated challenges in public health facilities of Oromiya special zone, Ethiopia**. I am visiting all health facilities in Oromiya special zone and your facility was part of this study. I would like to ask you a few questions regarding the practice of inventory control system.

Your participation is completely voluntary. You can refuse to answer questions and/or withdraw from the study at any time. All of the information collected is strictly confidential. No one other than the researcher will have access to your responses. Your identifiers such as your name and your health facility will not be revealed. The principal investigator will not refer to individual respondents or individual facilities in the report, but rather will describe the overall picture of all facilities. However, your honest answer to these questions will help us expand as well as improve the inventory control systems in particular and the health supply chain management systems in general.

Do I have your permission?


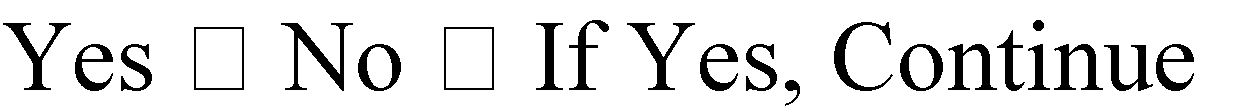

Supplement: Supplementary file 3 — Additional file 3. [file 12889_2021_12033_MOESM3_ESM.docx]
